# Effects of antimicrobial addition on lipid oxidation of rendered chicken fat

**DOI:** 10.1093/tas/txac011

**Published:** 2022-02-27

**Authors:** Janak Dhakal, Dalton Holt, Charles G Aldrich, Carl Knueven

**Affiliations:** 1 Department of Food Science and Technology, University of Nebraska-Lincoln, Lincoln, NE 68588, USA; 2 Department of Grain Science and Industry, Kansas State University, Manhattan, KS 66506, USA; 3 Jones-Hamilton Co., Walbridge, OH 43465, USA

**Keywords:** free fatty acid, organic acids, p-anisidine value, peroxide value, rendered chicken fat, sodium bisulfate

## Abstract

This study evaluated the effects of antimicrobial acidulant addition on lipid oxidation of rendered chicken fat. Chicken fat was untreated (control) or treated with either sodium bisulfate (SBS) or lactic acid (LA) at 0.5% w/w and incubated for 6 wk at 40 °C. Peroxide value (PV), p-anisidine value (AV), and free fatty acid (FFA) levels were measured at days 0 (D0), 1(D1), 3 (D3), 5 (D5), and 7 (D7), and weeks 2 (W2), 3 (W3), 4 (W4), 5 (W5), and 6 (W6). The FFA level of untreated-control fat was ~7% and remained consistent throughout the incubation until W6 (~8.5%; *P* < 0.05). The FFA values in SBS-treated fat were constant (range 7.25%–8.30%) throughout the incubation, whereas the FFA in LA-treated fat peaked at W5 (9.3%; *P* < 0.05). For the control fat, PVs were between 0.56 and 0.67 meq/100 g until W1 then declined. For the SBS-treated fat, the PVs remained low and similar to the control with the exception of a slight increase on W4 to 0.38 meqv/100 g (*P* < 0.05). In the LA-treated fat, the PV was greater than (*P* < 0.05) the control from W1 and increased to a peak on W5 (2.52 meq/100 g). The AV of control fat averaged 2.12 at D0 and increased through W2. In control and LA-treated fat, the AV values declined slightly thereafter, whereas SBS-treated fat increased (*P* < 0.05) to 10.28 on W5. This study indicates that when included at antimicrobial effective levels, LA may reduce the shelf-life of chicken fat, but SBS had a minimal effect over 6 wk of storage.

## INTRODUCTION

It is common to add fat topically to extruded pet food. This provides a source of added calories, essential fatty acids, flavor, and texture for the dog and cat. The fat also aids the absorption of fat-soluble vitamins (Bauer, 2006).

Most fats added to pet food are derived from animal sources as a function of rendering. Rendering is an effective means for the separation of fat from the remaining animal tissue through intense thermal processing (Romans et al., 2001), which is also an effective means for killing pathogenic organisms. However, recent indications suggest that following rendering the fat could be re-contaminated through handling, transport, and storage. Because fat is surface applied following the established kill step (extrusion in pet food), this represents a potential vector for the reintroduction of pathogens onto the food.

Commonly found in poultry products, *Salmonella* remains a significant economic and safety hazard in the food system (Batz et al., 2012). Although the application of heat during the rendering process is effective at inactivating most microorganisms, it does not provide protection for post-rendering re-contamination (Cochrane et al., 2016; Meeker and Hamilton, 2006). Recent work would suggest that acidulants such as lactic acid, phosphoric acid, or sodium bisulfate may provide residual preventive controls to pathogens introduced into fat (Dhakal et al., 2019). However, these acidulants could potentially impact the quality of the fat.

Lipid oxidation is a major concern regarding the nutritional quality of fat; whereby, lipid compounds are the least stable macronutrient. Degradation of fats and oils by oxidation has the potential of reducing digestibility, suppressing growth and development, and inducing oxidative stress of the animal. Few studies have directly documented the impact of feeding oxidized lipids in companion animals. The one publication of note by Turek et al. (2003) showed that both medium and high concentrations of secondary oxidation products in diets suppressed growth, markers of immune function, and bone formation in growing puppies relative to an unoxidized control. The concern herein was that the addition of acidulants to the fat might increase the rate of oxidation.

Currently, no published data are available evaluating antimicrobial acidulants such as lactic acid and sodium bisulfate in rendered chicken fat on lipid oxidation. Therefore, it was our objective to determine the effect of pathogen preventive acidulants, at their previously established doses (minimum inhibitory concentration), on oxidation of rendered chicken fat.

## MATERIALS AND METHODS

### Chicken Fat

Rendered chicken fat was procured from a regional poultry supplier within 24 h of the poultry slaughter (Simmons Foods, Silom Springs, AR). Samples from the rendering facility were shipped overnight to our research lab and the experiment was started within 24 h of receipt. The fat was treated with a commercial level of the natural antioxidant preservative blend (~1,600 ppm of liquid Naturox) in the rendering plant and was measured as 355.9 ppm of total tocopherols. The materials used were fresh and had no degradation issues such as off-color, off-odor, or rancidity. As the primary purpose of the study was to test the antimicrobial efficacy of the acidulants (SBS and lactic acid) and not as the source of antioxidant, every effort was made to simulate real industry conditions by adding the acidulants in market-ready rendered fat and then evaluate changes in the oxidation shelf-life of the fat.

### Treatment of Fat With Antimicrobials

An aliquot of chicken fat was transferred into 90 clean and grease-free plastic cups (Kroger 532-mL Textured Plastic Cups) and treated with two of the acidulants, 0.5% SBS, 0.5% LA, or distilled water (control). The acidulants were added to distilled water and added to the fat at a 3% final added moisture. This is a level consistent with the upper specification for commercial fat. The cups were filled with 100 mL of fat with 432 ml remaining headspace. All the sample cups were incubated at 40 °C for up to 6 wk. This temperature was selected to achieve sufficient fluidity for pipetting and corresponded to the melting point of the rendered chicken fat (~31–35 °C; Meeker and Hamilton, 2006). Subsamples for oxidative measurements were collected from the middle of the sample cups and included only chicken fat (not the added water) at the pre-determined time intervals.

### Shelf-Life Study of Rendered Chicken Fat

For the determination of free fatty acids (American Oil Chemists’ Society [AOCS] official method Ca 51-40 was used), 75 mL of hot neutralized alcohol was added to 7.05 g of well-mixed sample in an Erlenmeyer flask followed by 2 mL of phenolphthalein indicator. Shaking vigorously, the content was titrated using 0.1% potassium hydroxide (KOH) until the appearance of first permanent pink color of the same intensity as that of the hot neutralized alcohol which persisted for at least 30 s.

The peroxide value of the fat was determined (AOAC official method 965.33) by adding 30 mL of acetic acid: chloroform (3:2, v/v) to 3 g of sample in an Erlenmeyer flask with a glass stopper. After swirling to dissolve, 1 mL of saturated potassium iodide solution was added and mixed well. Then, after 1 min, 100 mL of distilled water was added to stop the reaction. After the addition of 1 mL of starch indicator, the mixture was titrated with 0.01N sodium thiosulfate under constant shaking until the blue color disappeared. A blank sample with 30 mL of acetic acid:chloroform and 1.0 mL of the starch solution was also prepared.

The p-anisidine value (AV) was determined (AOCS official method Cd 18-90) with 0.75 g of the fat sample transferred into a 25-mL volumetric flask and dissolved with 25 mL of isooctane. After dissolving for approximately 4–5 min, 5 mL of the sample was transferred into an aluminum foil-wrapped test tube followed by the addition of 1 mL of p-anisidine. After 10 min of reaction time, the sample was measured on a spectrophotometer at 350 nm. Both the sample (without p-anisidine) and reactions (with p-anisidine) were read separately.

### Statistical Analysis

The study was conducted as a completely randomized design with time intervals as the block. A total of 3 replications for each treatment was performed. At each of the 10 time points, a duplicate sample was tested. Statistical software (SAS; version 9.2) was used to analyze the data for the effects of treatments and within the time interval. Means were separated with a pairwise comparison and considered different at a Probability < 0.05.

## RESULTS AND DISCUSSION

Lipid oxidation is a series of chemical reactions involving oxygen which can be described in terms of oxidative rancidity, or deterioration of lipids, causing undesirable changes in color, taste, odor, palatability, and destruction of essential fatty acids. Oxidation rates in lipids are largely a function of the structure of the fatty acid chains (Kilcast and Subramaniam, 2000; Osawa et al., 2008). Poultry fats contain high percentages of unsaturated fatty acids (ranging 57%–75%) with linoleic acid (C18:2) accounting for as much as 20% of the total fatty acid profile, much higher than that of beef tallow and pork lard (Hu and Jacobsen, 2016). Unsaturated fatty acids contain reactive double bonds between some of their carbon atoms, and this results in higher susceptibility to oxidation. Numerous analytical methods exist for the measurement of lipid oxidation, each with their own value and limitations (Hu and Jacobsen, 2016; Gray, 2015).

**Figure 1.**
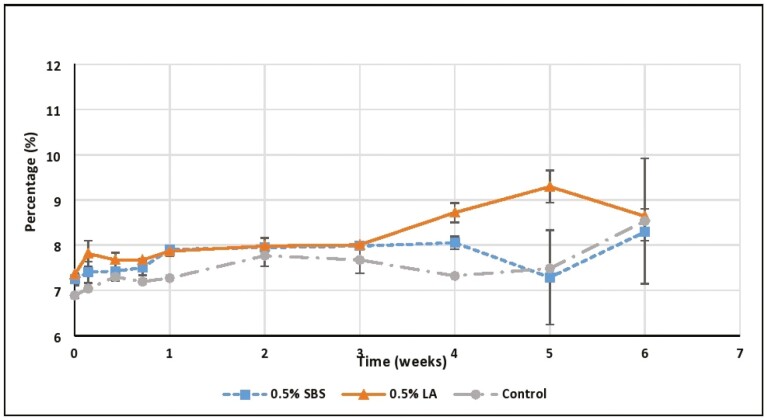
Effects of acidulants in the free fatty acid levels of rendered chicken fat evaluated over the period of 6 wk at 40 °C. SBS, sodium bisulfate; LA, lactic acid; Control, no acidulant. ∗LA > SBS, Control (*P* < 0.05).

Lipid oxidation is complex and involves multiple reactions (Osawa et al., 2008). The measure of free fatty acids (FFA) and peroxide values (PV) serve as early indicators of the potential for fats to oxidize. The FFA value measures the concentration of free fatty acids cleaved from the triacylglyceride molecules by the hydrolytic breaking of the ester bonds between the fatty acids and the glycerol backbone (Miller, 2010). The percentage FFA in this study was expressed as a percentage (in weight) of oleic acid-based on titration with a standard solution of KOH using phenolphthalein as the indicator (AOCS, 2009). The free fatty acid concentration of the rendered chicken fat used in this study was approximately 6.88% on initiation of the study and remained consistent through week 5; however, on week 6, FFA increased slightly to 8.53% (*P* < 0.05; Figure 1). The hydrolysis of fat can occur due to the enzymatic (lipase) hydrolysis before rendering, or through acid or steam hydrolysis after rendering. The increase in the FFA over time in the LA added chicken fat could be due to the acid hydrolysis of fat by lactic acid leading to more free fatty acid production. This finding is also related to the report of Tadesse et al. (2017) who also observed a constant increase in the FFA over the incubation time with no regular pattern of increase. Osawa et al. (2008) reported that the FFA level in dry pet food samples increased during the storage period. They reported a range of 4.6% to 28.0% FFA (as oleic acid equivalents) in the pet food samples. The FFA percentage, as well as the range in our study, was smaller compared to their finding which could be dependent on the fat source and storage time before analysis. The normal range of FFA in livestock and poultry feed can vary widely. For example, a review by Shurson et al. (2015) reported that FFA values of lipid surveyed from local feed mills varied from 5.8% to 51.6%.

**Figure 2.**
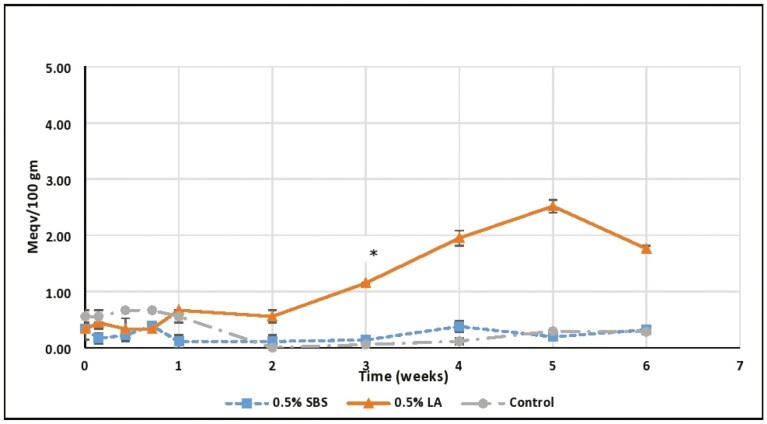
Effects of acidulants in the peroxide value of rendered chicken fat evaluated over the period of 6 wk at 40 °C. SBS, sodium bisulfate; LA, lactic acid; Control, no acidulant. ∗LA > SBS, Control (*P* < 0.05) (but, on week 1 it is LA, SBS > Control).

Peroxide value measures the primary oxidative products of fat (Karakaya and Şimşek, 2011). In the control fat, the PV ranged from 0 to 0.67 meq/100 g fat throughout the storage period (Figure 2). The values were in a range of 0.56–0.67 meqv/100 g fat for the first week before it declined. This decline may be due to primary oxidation products decomposing to form secondary oxidation products as storage time increased. A similar mechanism was observed by Liu et al. (2019) in peanut butter when stored at 35 °C. The PV value of chicken fat stored for 7 d at 4 °C was 0.215 meq/100 g of fat in the study of Shantha and Decker (1994). This lower PV value compared to our study may be due to the lower storage temperature (4 °C vs. 40 °C in the current study). The PV values in the SBS treated fat also remained in a close range (0.11–0.39 meq/100 g of fat) with a higher value (*P* < 0.05) on day 5 and week 4 of the sampling. The reason for these elevated values is not immediately obvious. The continuous increase in the PV value up to day 5 in control and SBS-treated fat in our study may be similar to the findings of Tadesse et al. (2017), wherein the PV value in animal butter continuously increased during the storage period (72 h) when stored at 25 °C and 65 °C. However, in the LA-treated chicken fat, the PV values increased over the storage period until 5 wk and then began to decline on week 6. The highest PV value (2.53 meq/100 g of fat) was recorded on week 5. The rising PV values prior to week 6 may be the acidulant (lactic acid) causing more lipid oxidation to form peroxides before breaking down to secondary oxidation products. The PV values in the LA-treated and control fat increased linearly (*P* < 0.05) over time. A PV range in lipids of 10–20 meq/kg fat is considered normal (Connell, 1975). The reported levels of PV in livestock and poultry feed have ranged from 0.4 to 7.3 mEq/kg (Shurson et al., 2015). De Marchi et al. (2018) reported a PV range of 2.2–94.10 mEq O2/kg fat across 208 different extruded dog foods. It is important to note that PV and FFA levels are not a determination of rancidity but rather a quality measure dictated by the purchaser as an indicator of initial oxidation.

**Figure 3.**
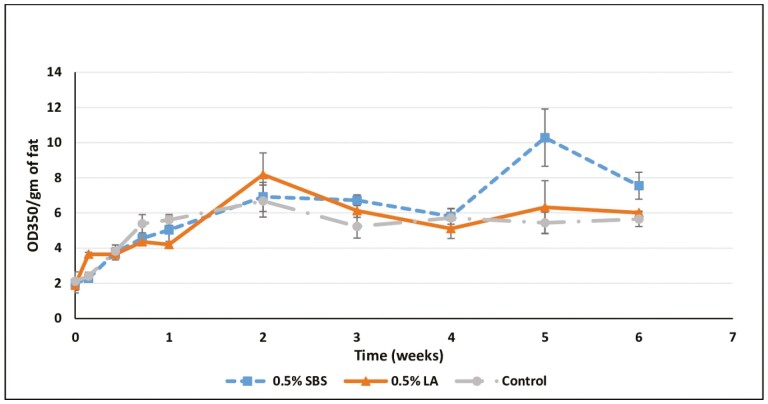
Effects of acidulants in the anisidine values of rendered chicken fat evaluated over the period of 6 wk at 40 °C. SBS, sodium bisulfate; LA, lactic acid; Control, no acidulant. ∗LA > SBS, Control (*P* < 0.05).

The second stage of oxidation occurs as hydroperoxides are cleaved to form carbonyl compounds such as aldehydes, which can be measured as non-volatile secondary oxidation products by the AV method (Steele, 2004). It is these secondary compounds of oxidation that represent lipid rancidity that impacts food quality. Aldehydes, ketones, and alcohols produced as secondary oxidation compounds produce off-tastes and off-smells (Hu and Jacobsen, 2016) affecting sensory properties of the food as well as the toxic components affecting animal health (Turek et al., 2003). It is important to note that initially peroxide values increase during lipid oxidation, but as secondary compounds are formed these primary values drop, so it is essential to consider these results in tandem to aid in deciphering what may be occurring at the time of measurement. The AV values for the control fat ranged between 2.11 and 6.68. The LA-treated fat had AV values between 1.89 on day 0 and 8.18 on week 2, whereas the SB-treated fat had AV values of 2.00 on day 0 to 10.28 on week 5 (Figure 3). For a good quality fat or oil, the AV values should be lower than 10 (List et al., 1974). In our study, both the acidulant-treated fats and control fat had AV values at or below this threshold, with the single exception of a 10.28 value observed for SBS-treated fat on week 5. The higher AV value indicates declining quality for the fat. Tadesse et al. (2017) reported that the AV values of animal butter increased during storage at 65 °C in a manner similar to our findings at 40 °C storage. Christensen and Holmer (1996) reported that the increase in the AV values of fats and oils was a function of storage time and temperature. The time effect and the interaction of the main effects were significant (*P* < 0.05). Both the acidulant-treated samples, as well as the control, showed a linear (*P* < 0.05) increase in the AV value over time.

**Figure 4.**
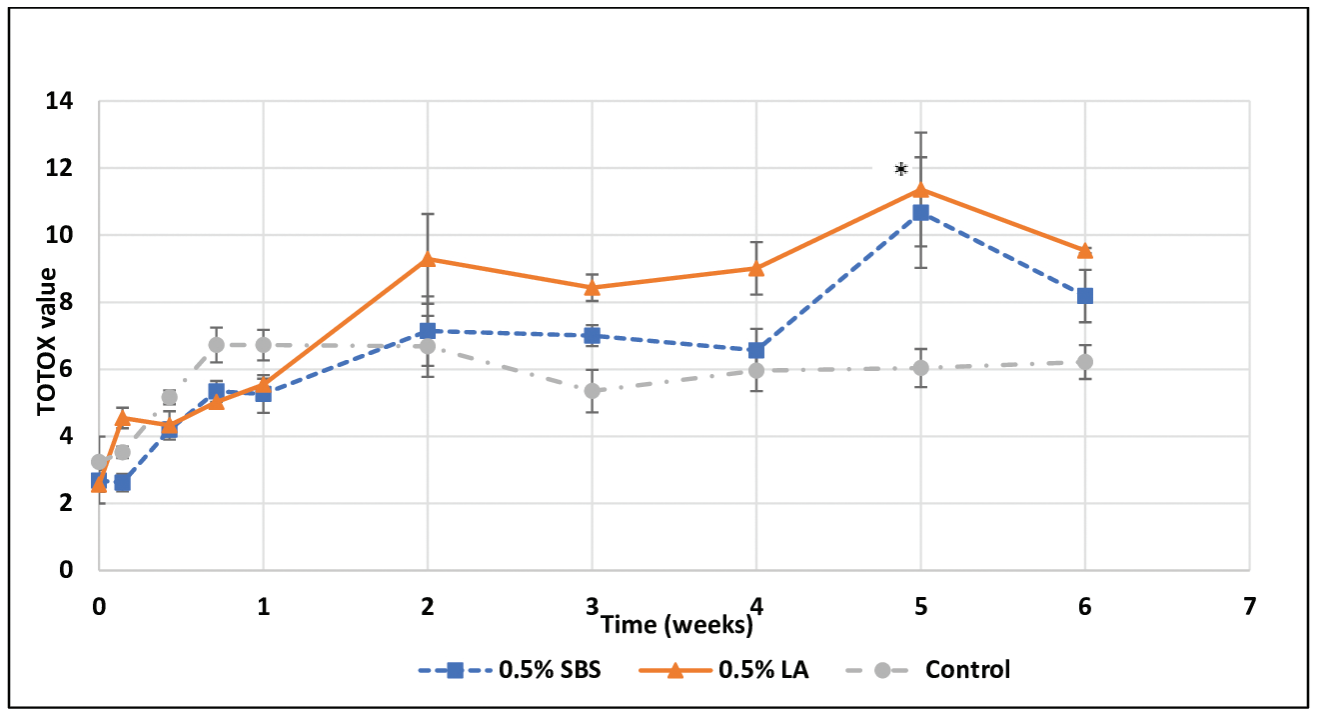
Effects of acidulants in the TOTOX values of rendered chicken fat evaluated over the period of 6 wk at 40 °C. SBS, sodium bisulfate; LA, lactic acid; Control, no acidulant. ∗LA, Control > SBS (*P* < 0.05).

In the fats and oil industry, the TOTOX value has been proposed as a means to combine the anisidine value and the peroxide value (Shahidi et al., 2002; O’ Keefe et al., 2010). The TOTOX value provides a broad accounting for the history of an oil or fat, but it “does not have any sound scientific basis because it combines variables with different dimensions” according to Shahidi et al. (2002). The TOTOX value is calculated by adding the AV value twice the PV value. There was a linear increase (*P* < 0.05) in TOTOX values over time for all treatments. Individually, the TOTOX values of control fat after 1 wk of storage remained constant over the storage period, whereas the TOTOX values for the acidulant treated chicken fats increased throughout the storage time with a maximum TOTOX of 10.67 on week 5 for SBS treated fat and 11.36 on week 5 for LA-treated fat (Figure 4) and differed (*P* < 0.05) from the control (TOTOX of 6.0).

## CONCLUSION

This study demonstrated that the FFA of fat rose slightly over the 6 wk regardless of the acidulant treatments. The addition of LA increased the PV of the chicken fat and SBS led to a slight increase in AV in the last 2 wk of the study. Taken in combination the acidulant treatment each led to a rise in TOTOX values for the fat by weeks 5 and 6. This would indicate that the uses of acidulants for pathogen control are stable to oxidation for at least 4 wk and that LA may have a greater impact on primary oxidation products (PV), whereas SBS has more impact on secondary oxidation products (AV) beyond 4 wk of storage. There may be limitations to these findings because the experiment was conducted in a “bulk oil” model, and the results might be amplified if applied in a thin layer to pet foods where exposure to air would be greater. Future studies should consider a longer storage duration. The immediate concern was during bulk storage of fats from time of production to application on pet food. Most of which would be consumed within 10 d to 2 wk. The evaluation of the Oxidative Stability Index (OSI) was considered but rejected due to the intention for the evaluation of fat degradation or quality decline rather than capacity for storage stability. For our future evaluations, a sensory analysis with trained panelists, coupled with analysis for volatiles and animal acceptance, might be worthwhile. Presuming that these animal and human factors are acceptable, then the longer-range work should consider the effects on pet food kibbles coated with rendered chicken fat treated with acidulants. In conclusion, although minor changes in oxidative rancidity were observed over 6 wk, due to antimicrobial acidulant, the chicken fat samples remained within acceptable levels for use in pet food applications.
